# RNA modification-related variants in genomic loci associated with body mass index

**DOI:** 10.1186/s40246-022-00403-1

**Published:** 2022-07-25

**Authors:** Jingyun Wu, Mimi Wang, Limin Han, Huan Zhang, Shufeng Lei, Yonghong Zhang, Xingbo Mo

**Affiliations:** 1 Jiangsu Key Laboratory of Preventive and Translational Medicine for Geriatric Diseases, Department of Epidemiology, School of Public Health, Soochow University, 199 Renai Road, Suzhou, 215123 Jiangsu People’s Republic of China; 2 Jiangsu Key Laboratory of Preventive and Translational Medicine for Geriatric Diseases, Center for Genetic Epidemiology and Genomics, School of Public Health, Soochow University, Suzhou, China

**Keywords:** Body mass index, RNA modification, Genome-wide association study, Gene expression

## Abstract

**Background:**

Genome-wide association studies (GWASs) have identified hundreds of loci for body mass index (BMI), but functional variants in these loci are less known. The purpose of this study was to identify RNA modification-related SNPs (RNAm-SNPs) for BMI in GWAS loci. BMI-associated RNAm-SNPs were identified in a GWAS of approximately 700,000 individuals. Gene expression and circulating protein levels affected by the RNAm-SNPs were identified by QTL analyses. Mendelian randomization (MR) methods were applied to test whether the gene expression and protein levels were associated with BMI.

**Results:**

A total of 78 RNAm-SNPs associated with BMI (*P* < 5.0 × 10^–8^) were identified, including 65 m^6^A-, 10 m^1^A-, 3 m^7^G- and 1 A-to-I-related SNPs. Two functional loss, high confidence level m^6^A-SNPs, rs6713978 (*P* = 6.4 × 10^–60^) and rs13410999 (*P* = 8.2 × 10^–59^), in the intron of *ADCY3* were the top significant SNPs. These two RNAm-SNPs were associated with *ADCY3* gene expression in adipose tissues, whole blood cells, the tibial nerve, the tibial artery and lymphocytes, and the expression levels in these tissues were associated with BMI. Proteins enriched in specific KEGG pathways, such as natural killer cell-mediated cytotoxicity, the Rap1 signaling pathway and the Ras signaling pathway, were affected by the RNAm-SNPs, and circulating levels of some of these proteins (ADH1B, DOCK9, MICB, PRDM1, STOM, TMPRSS11D and TXNDC12) were associated with BMI in MR analyses.

**Conclusions:**

Our study identified RNAm-SNPs in BMI-related genomic loci and suggested that RNA modification may affect BMI by affecting the expression levels of corresponding genes and proteins.

**Supplementary Information:**

The online version contains supplementary material available at 10.1186/s40246-022-00403-1.

## Background

In recent decades, obesity, a metabolic disease, has become globally prevalent. The number of people suffering from obesity nearly tripled from 1980 to 2016[1]. With its overwhelming effects on national health and the social economy, the epidemic of obesity poses a great challenge to the healthcare system[2]. Body mass index (BMI) is used to evaluate overweight or obesity. According to the standard established by the World Health Organization, 25 kg/m^2^ ≤ BMI < 30 kg/m^2^ is characterized as overweight, and BMI ≥ 30 kg/m^2^ is obesity. Approximately 34.3% and 16.4% of adults are overweight and obese, respectively, and the prevalence of obesity is affected by age, urbanization and the level of education[3]. Obesity is a common risk factor for cardiovascular disease, hypertension, type 2 diabetes and some types of cancer[4–6].

Obesity is the result of a combination of many factors, including environmental factors and the expression of some genes, and is heritable[7]. The heritability of obesity is generally 40–70%[8]. Genome-wide association studies (GWASs) have identified a large number of BMI-associated loci. In recent years, with the rapid development of genetic epidemiological approaches, particularly GWASs, the number of SNPs associated with BMI has increased substantially[9]. However, risk factors involved in the pathway of the regulation of genetic variants on BMI and the functional roles of the GWAS-identified genes in the development of obesity are still unclear. Elucidation of their biological functions can be very useful for the translation of GWAS signals into causal mechanisms and clinical applications.

In recent years, studies have focused on the role of epigenetics in metabolism-related diseases such as obesity, and a link between epigenetic modification and metabolic health in humans has been proposed [10, 11]. Studies related to the modification of protein-coding and noncoding RNAs are rapidly accumulating. To date, over 170 different types of RNA modifications have been identified in various RNA molecules. This number is growing with the improvement of technical approaches. N6-methyladenosine (m^6^A), which is one of the most important modifications in eukaryotic messenger RNAs, plays a crucial role in various biological processes of living organisms, such as gene expression regulation[12–14]. Studies have shown that RNA molecules are unstable and susceptible to dynamic, reversible chemical modifications, which may alter gene expression and affect RNA function [15, 16]. Single nucleotide polymorphisms affecting RNA modification (RNAm-SNPs) play key roles in many aspects of RNA metabolism and have recently been linked to many metabolic diseases [17–21].

At present, it is not clear how RNAm-SNPs affect BMI. This study posited that the GWAS-identified BMI-associated loci contain RNAm-SNPs, which may be important functional variants, and that the RNAm-SNPs affect BMI by altering gene expression at the RNA or protein level. Therefore, this study first distinguished RNAm-SNPs from other types of SNPs in BMI-associated genomic loci. Then, the impacts of RNAm-SNPs on gene expression were evaluated in quantitative trait locus (QTL) studies, including RNA expression QTL (eQTL) and circulating protein levels QTL (pQTL), to support the functionality of the RNAm-SNPs. By applying Mendelian randomization (MR) analysis methods, the associations between gene expression and circulating protein levels and BMI were examined, and thus, potential novel risk factors for obesity were identified.

## Materials and methods

### Determination of RNAm-SNPs for BMI

The RNAm-SNP information is available in the RMVar database and can be accessed at http://rmvar.renlab.org/download.html. RMVar, a database of functional variants involved in RNA modification, contains more than 1.6 million RNA modification-related variants for nine types of RNA modifications, including m^6^A (N6-adenosine methylation), m^5^C (5-methylcytidin), A-to-I RNA editing, Nm (ribose 2'-O-methylation), Ψ (pseudouridine), m^7^G (N7-methylguanosine), m^1^A (N1-adenosine methylation), m^5^U (5-methyluridine) and m^6^Am (N6,2'-O-dimethyladenosine) [17]. Compared with the m6Avar database (older version), not only are the numbers and categories of RNA modifications increased, but the information is also annotated more comprehensively. Due to different acquisition methods, the confidence levels of these RNAm-SNPs are classified as high, medium and low. According to the rs ID numbers of the RMVar and BMI GWAS databases, RNAm-SNPs associated with BMI were screened out (*P* < 5.0 × 10^–8^).

In this study, RNAm-SNPs associated with BMI were obtained by integrating summary data from a BMI GWAS with information from the RMVar database. Summary statistics of associations between more than 10 million SNPs and BMI have been collected from a BMI GWAS dataset (http://cnsgenomics.com/data.html), which was assessed on approximately 700,000 individuals of European ancestry, including ~ 250,000 participants from the Genetic Investigation of ANthropometric Traits (GIANT) consortium study and ~ 450,000 participants from the UK Biobank[9]. The ~ 250,000 participants from the GIANT study were European-descent individuals[22]. The UK Biobank included subjects of European, African and South Asian ancestries[23], but in this GWAS, the analysis was restricted to 456,426 participants of European ancestry[9]. Replication analysis in the East Asian population for the identified associations was performed. Data for the replication analysis were obtained from a GWAS of BMI in 158,284 Japanese people[24]. Summary statistics of associations between 5,961,600 SNPs and BMI were downloaded at http://jenger.riken.jp/en/result.

### eQTL analysis for BMI-associated RNAm-SNPs

As an important epigenetic modification, gene expression regulation is one of the most important roles of RNA modifications. RNAm-SNPs may affect BMI by regulating the expression levels of mRNAs. Therefore, eQTL analysis was performed to discover the association between RNAm-SNPs and mRNA expression levels in different types of cells and tissues. Generally, eQTLs can be divided into two categories: *cis*-eQTLs and *trans*-eQTLs. This study mainly focused on *cis*-eQTL effects. The *cis*-eQTL identified in this study means that the RNAm-SNP is located in the genomic region of a gene and was associated with the expression levels of its host genes. The data required for analysis can be obtained via the HaploReg browser (http://archive.broadinstitute.org/mammals/haploreg/haploreg.php), which contains eQTL data from 13 studies performed in different human tissues and cells[25].

The SMR (summary data–based Mendelian randomization) approach [26] was applied to identify the associations between gene expression levels and BMI by integrating summary-level data from eQTL studies with summary-level data from the BMI GWAS. We evaluated the associations between gene expression levels in six relevant tissues (subcutaneous and visceral omentum adipose tissues, skeletal muscle, whole blood, pancreas and thyroid) and BMI. The data needed for SMR analysis were collected from the BMI GWAS dataset[9] and eQTL data from the GTEx project[27]. The eQTL summary dataset of the nine tissues in SMR file format can be downloaded at http://cnsgenomics.com/software/smr/#DataResource. The SMR package (version 0.712) is available at http://cnsgenomics.com/software/smr/index.html. It is a command line program running under the Windows system. Default parameters of SMR were used in the analysis, and results with *P* < 5.0 × 10^–6^ were considered significant. In addition, the HEIDI test was employed to test for heterogeneity in SMR association statistics.

### pQTL analysis for BMI-associated RNAm-SNPs

RNAm-SNPs may also affect BMI by regulating gene expression at the protein level. Circulating proteins play important roles in many biological processes and are important therapeutic targets. Therefore, pQTL analysis was further applied to identify circulating proteins associated with the identified RNAm-SNPs. The data used for pQTL analysis were collected from the INTERVAL pQTL study[28]. This study enrolled 3,301 individuals of European descent and examined the associations between 10.6 million imputed autosomal variants and circulating levels of 2,994 proteins (http://www.phpc.cam.ac.uk/ceu/proteins/).

### Functional enrichment analysis

KEGG (Kyoto Encyclopedia of Genes and Genomes; https://www.kegg.jp/kegg/) is a collection of databases dealing with genomes, diseases, biological pathways, drugs and chemical materials that can help us understand the functional interpretation of genes and their products as a whole network. Genetic ontology (GO; http://www.geneontology.org) is a bioinformatics resource that uses the ontology to represent biological knowledge and provides information about gene function. The GO project is a powerful tool for a comprehensive description of functional genomics. It is a common resource that describes the various roles of genes in biological systems, including biological processes, molecular functions and cellular components. DAVID, an online feature annotation tool[29], was used to perform the functional enrichment analysis. It can be performed through https://david.ncifcrf.gov/website.

### MR analysis of proteins

To obtain further supporting evidence for proteins identified in pQTL analysis, we employed four MR analysis methods, including the weighted median[30], inverse-variance weighted (IVW) [31], MR-Egger[32] and MR pleiotropy residual sum and outlier (MR-PRESSO)[33], to test for potential causal relationships between circulating protein levels and BMI. The IVW method combines the ratio estimates from each IV in a meta-analysis model[31]. If the associations with circulating protein levels were to lead to horizontal pleiotropy, the intercept from MR-Egger would be expected to differ from zero[32]. Weighted median estimation can provide a consistent assessment if more than 50% of the weights for the SNPs come from valid SNPs[30]. We also applied the MR-PRESSO method to detect horizontal pleiotropy and obtain outlier-corrected causal estimations [33]. The outlier test in MR-PRESSO is the procedure to test for the MR assumption of no pleiotropy.

The data used in these MR analyses were the pQTL and GWAS data described above. Data required in the analyses (i.e., the SNP rs number, beta values, standard errors and *P* values) were extracted from the pQTL study and GWAS datasets described above. In the pQTL summary data, SNPs with *P* values less than 5.0 × 10^–6^ were selected as potential instrumental variables. To select independent instrumental variables, we applied the “clump_data” function in the R package TwoSampleMR to clump SNPs within 10,000 kb with the criteria of linkage disequilibrium r^2^ < 0.01 based on data from Europeans from the 1000 Genomes project. Then, we used the “merge” function of the R program to incorporate the summary data of the instrumental variables into a specific file (an ordinary document with 5 columns of the SNP rs number, beta values for protein, standard errors for protein, beta values for BMI and standard errors for BMI) for each protein-trait pair. After that, the effect allele of each instrumental variable in the BMI GWAS and pQTL studies was manually checked for consistency.

The weighted median, IVW and MR-Egger analyses were performed by using the MendelianRandomization R package[34]. The source code and documents for MR-PRESSO are available at https://github.com/rondolab/MR-PRESSO. In the MR-PRESSO analysis, parameters were left to their default values.

## Results

### BMI-associated RNAm-SNPs

First, a total of 4,820 RNAm-SNPs were selected by integrating the BMI GWAS and RMVar database. According to *P* < 5.0 × 10^–8^, 78 BMI-associated RNAm-SNPs containing four types of RNA modifications were identified, including 65 m^6^A-, 10 m^1^A-, 3 m^7^G- and 1 A-to-I-related SNPs (Additional file 1: Table S1). Among them, the 3'-UTR SNP rs17771942 (*P* = 7.4 × 10^–10^) in *SOCS5* was related to both m^6^A and m^1^A.

**Fig. 1:**
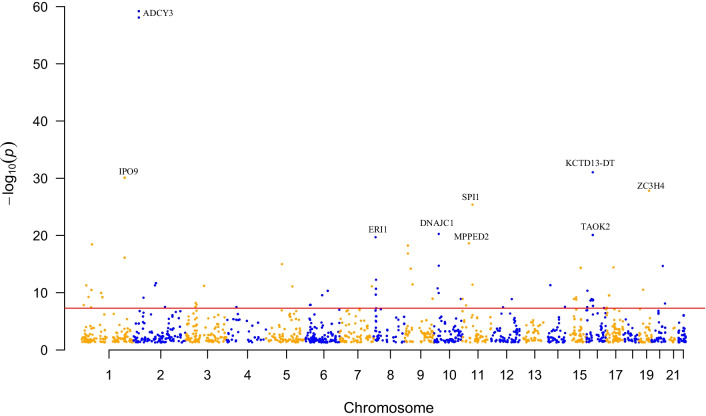
Genome-wide distribution of the identified BMI-associated m^6^A-SNPs. This Manhattan plot shows the associations between m^6^A-SNPs and BMI. The x-axis indicates chromosome positions. The y-axis indicates -log_10_*P* values of the associations. The *P* value information was extracted from the summary dataset of the BMI GWAS. The red line indicates the significance level of 5.0 × 10^–8^. Genes containing the top 10 most significant m^6^A-SNPs were annotated

**Fig. 2:**
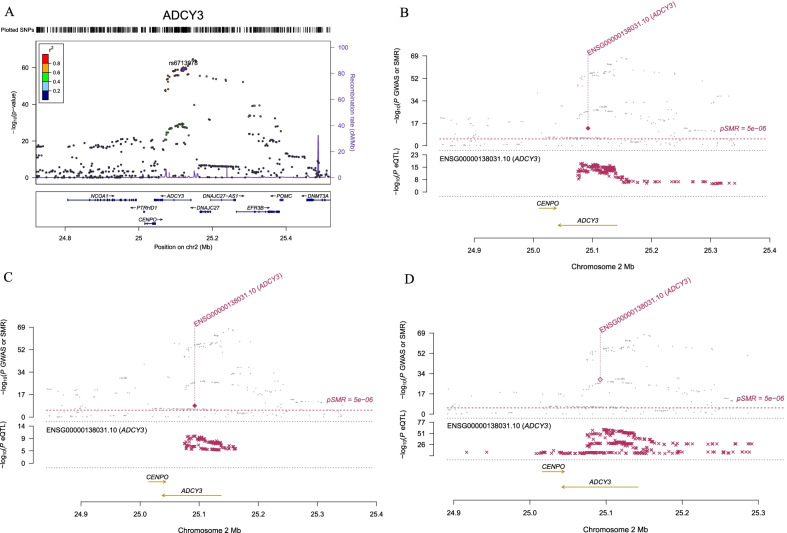
Association between the *ADCY3* gene and BMI. **A** The m^6^A-SNPs rs6713978 and rs13410999 in the *ADCY3* gene were significantly associated with BMI; SNPs in *ADCY3* were strongly associated with the expression level of *ADCY3*, and the expression levels of the *ADCY3* gene in subcutaneous **B** and visceral omentum adipose tissues **C** and whole blood cells **D** were significantly associated with BMI

The number of BMI-associated SNPs related to m^6^A was the largest (Fig. 1). Most (n = 53) of them were located in protein-coding genes (81.5%); 10 were located in lncRNAs (MIR137HG, LINC01114, LOC105377876, LINC00599, LOC105369928, MEG9, KCTD13-DT, ZNF747-DT, FBXL19-AS1 and LINC01524), and 2 were located in pseudogenes (SERBP1P3 and RPL27AP5). Among the 65 m^6^A-SNPs, 25, 15 and 25 RNAm-SNPs belonged to the high confidence, medium confidence and low confidence levels, respectively; 50 were functional loss, and 15 were functional gain. For the 53 m^6^A-SNPs located in protein-coding genes, 16 (30.8%) were in the 3'-UTR, 2 (3.1%) were in the 5'-UTR, 20 (36.9%) were intronic and 15 (32.3%) were exonic, among which 13 missense and 8 synonymous variants were found (Additional file 1: Table S1). The top 10 most significant BMI-associated SNPs related to m^6^A methylation are presented in Table 1. Two functional loss, high confidence level m^6^A-SNPs, rs6713978 (*P* = 6.4 × 10^–60^) and rs13410999 (*P* = 8.2 × 10^–59^), in the intron of *ADCY3* were the top significant SNPs (Fig. 1, Fig. 2A), followed by the functional gain m^6^A-associated SNP rs12716973 (*P* = 8.9 × 10^–32^) in the lncRNA KCTD13-DT. Replication analysis in the East Asian population identified associations between 11 of the 65 m^6^A-SNPs and BMI, including the associations between rs6713978 (*P* = 3.5 × 10^–10^) and rs13410999 (*P* = 2.6 × 10^–7^) in *ADCY3* and BMI (Additional file 1: Table S2).

**Table 1:** Top 10 most significant BMI-associated SNPs related to m^6^A methylation

SNP	Chromosome	Position	Gene	Gene type	Gene region	Effect allele	Other allele	Frequency	beta	se	*P* value	Confidence level	Modification function
rs6713978	2	24,897,982	ADCY3	Protein coding	intron	T	C	0.5593	−0.0319	0.002	6.40E−60	m6A-Label-seq: (High)	Functional Loss
rs13410999	2	24,875,070	ADCY3	Protein coding	intron	T	C	0.5548	−0.0316	0.002	8.20E−59	m6A-Label-seq: (High)	Functional Loss
rs12716973	16	29,926,333	KCTD13-DT	lncRNA	exon	A	G	0.5435	0.0231	0.002	8.90E−32	Prediction: (Low)	Functional Gain
rs8024	1	201,876,446	IPO9	Protein coding	3’-UTR	A	C	0.337	0.0237	0.0021	8.00E−31	miCLIP: (High)	Functional Loss
rs7250850	19	47,082,263	ZC3H4	Protein coding	CDS	C	G	0.7006	0.024	0.0022	1.50E−28	Prediction: (Low)	Functional Gain
rs1057233	11	47,354,898	SPI1	Protein coding	3’-UTR	A	G	0.6743	−0.0222	0.0021	4.10E−26	MeRIP-seq: (Medium)	Functional Loss
rs4146689	10	21,987,890	DNAJC1	Protein coding	intron	A	C	0.2778	0.0208	0.0022	5.30E−21	m6A-Label-seq: (High)	Functional Loss
rs4077410	16	29,986,877	TAOK2	Protein coding	CDS	A	G	0.4751	−0.0183	0.002	8.00E−21	MeRIP-seq: (Medium)	Functional Loss
rs2979247	8	9,031,438	ERI1	Protein coding	3’-UTR	A	G	0.5807	−0.0185	0.002	2.00E−20	Prediction: (Low)	Functional Loss
rs13642	11	30,410,661	MPPED2	Protein coding	3’-UTR	A	T	0.6373	0.0183	0.002	2.30E−19	Prediction: (Low)	Functional Gain

The 10 m^1^A-SNPs associated with BMI at *P* < 5 × 10^–8^ are all located in protein coding genes and are all functional loss. Among them, 6 belonged to the high confidence level and 4 belonged to the medium confidence level; 3 were in the 3'-UTR, 3 were intronic, and 4 were exonic (2 missense and 2 synonymous) (Table 2, Additional file 1: Table S1). The most significant SNP was the intronic SNP rs3803286 (*P* = 1.7 × 10^–20^) in *TRAF3*, followed by rs1051436 (*P* = 3.9 × 10^–12^) in the 3'-UTR of *CGGBP1*. An association between rs227584 in *HROB* and BMI was also found in the East Asian population (Additional file 1: Table S2).

**Table 2:** The significant BMI-associated SNPs related to m^1^A modification

SNP	Chromosome	Position	Gene	Gene type	Gene region	Effect allele	Other allele	Frequency	beta	se	*P* value	Confidence level	Modification function
rs17771942	2	46,762,123	SOCS5	Protein coding	3’-UTR	A	T	0.91347	−0.0208	0.0034	7.40E−10	m1A-quant-seq: (High)	Functional Loss
rs1051436	3	88,052,380	CGGBP1	Protein coding	3’-UTR	T	C	0.1474	−0.0187	0.0027	3.90E−12	m1A-quant-seq: (High)	Functional Loss
rs1569228	3	136,839,917	SLC35G2	Protein coding	intron	T	C	0.3137	−0.0129	0.0021	7.80E−10	MeRIP-seq: (Medium)	Functional Loss
rs1047643	8	11,802,853	FDFT1	Protein coding	CDS	T	C	0.8391	0.0143	0.0026	3.40E−08	MeRIP-seq: (Medium)	Functional Loss
rs10902221	11	802,379	PIDD1	Protein coding	CDS	T	C	0.4825	−0.0118	0.002	1.80E−09	m1A-quant-seq: (High)	Functional Loss
rs3803286	14	102,780,133	TRAF3	Protein coding	intron	A	G	0.3431	0.0193	0.0021	1.70E−20	MeRIP-seq: (Medium)	Functional Loss
rs9479	15	74,036,235	PML	Protein coding	intron	A	G	0.5001	−0.0113	0.002	8.50E−09	m1A-quant-seq: (High)	Functional Loss
rs227584	17	44,148,179	C17orf53	Protein coding	CDS	A	C	0.6945	−0.0123	0.0021	9.50E−09	m1A-quant-seq: (High)	Functional Loss
rs8044	19	49,150,749	HRC	Protein coding	3’-UTR	T	G	0.409	0.0127	0.002	2.40E−10	MeRIP-seq: (Medium)	Functional Loss
rs14194	21	39,177,540	PSMG1	Protein coding	CDS	T	C	0.6435	0.0116	0.002	1.20E−08	m1A-quant-seq: (High)	Functional Loss

For the m^7^G modification, only 3 m^7^G-SNPs associated with BMI (*P* < 5.0 × 10^–8^) were identified (Additional file 1: Table S1), including the missense mutation rs11545169 (*P* = 3.3 × 10^–9^) in *PSMD2*, rs11596235 (*P* = 2.7 × 10^–8^) in the 3'-UTR of *SUFU*, and the synonymous mutation rs2270576 in *SNF8* (*P* = 1.4 × 10^–13^). In addition, one functional loss A-to-I modification-related SNP, rs2577951, in an intron of *MGA* was identified (*P* = 4.2 × 10^–9^). An association between rs11596235 in *SUFU* and BMI was also found in the East Asian population (Additional file 1: Table S2).

### Gene expression associated with BMI

We investigated whether the RNAm-SNPs were associated with gene expression. eQTL analysis was performed for the 78 identified RNAm-SNPs associated with BMI (*P* < 5.0 × 10^–8^). According to the HaploReg database, most of these RNAm-SNPs (84.6%) showed eQTL effects in different cells or tissues, and *cis*-eQTL signals were found for 39 RNAm-SNPs. Among these 39 *cis*-acting RNAm-SNPs, 32 were related to m^6^A, 6 were related to m^1^A and 1 was related to m^7^G. For example, the m^6^A-SNPs rs13410999 and rs6713978 in *ADCY3* were associated with the expression levels of *ADCY3* in subcutaneous adipose tissue (*P* = 3.97 × 10^–7^ and 1.11 × 10^–6^, respectively) and whole blood cells (*P* = 1.91 × 10^–57^ and 1.62 × 10^–52^, respectively). A total of 31 RNAm-SNPs showed eQTL effects in adipose tissues (Additional file 1: Table S3), and 11 of them in *GPBP1L1*, *EVI5*, *ADCY3*, *IP6K2*, *SERBP1P3*, *PIDD1*, *RPAIN*, *ZSWIM7*, *HAPLN4* and *PSMG1* were *cis*-acting RNAm-SNPs. The two top significant *cis*-acting RNAm-SNPs in adipose tissues were rs14194 in *PSMG1* and rs10902221 in *PIDD1* (*P* = 1.04 × 10^–16^ and 9.80 × 10^–14^, respectively), which were associated with m^1^A methylation.

In SMR analysis, we found that the expression levels of 12 genes in the six relevant tissue types were significantly associated with BMI (*P* < 5.0 × 10^–6^) (Additional file 1: Table S4). The number of significant associations found in each tissue was 6 in adipose tissue, 7 in skeletal muscle, 4 in whole blood cells, 4 in the thyroid and 2 in the pancreas. We found that the expression levels of some key obesity susceptibility genes were significantly associated with BMI in related tissues. For example, the expression levels of the *ADCY3* gene were significantly associated with BMI in subcutaneous (*P* = 3.25 × 10^–14^) (Fig. 2B) and visceral omentum adipose tissues (*P* = 1.86 × 10^–9^) (Fig. 2C) and whole blood cells (*P* = 3.62 × 10^–30^) (Fig. 2D). The results of the eQTL and SMR analyses suggested that the RNAm-SNPs rs6713978 and rs13410999 may affect *ADCY3* gene expression in these tissues and then affect obesity risk.

### Circulating proteins related to the RNAm-SNPs

**Fig. 3:**
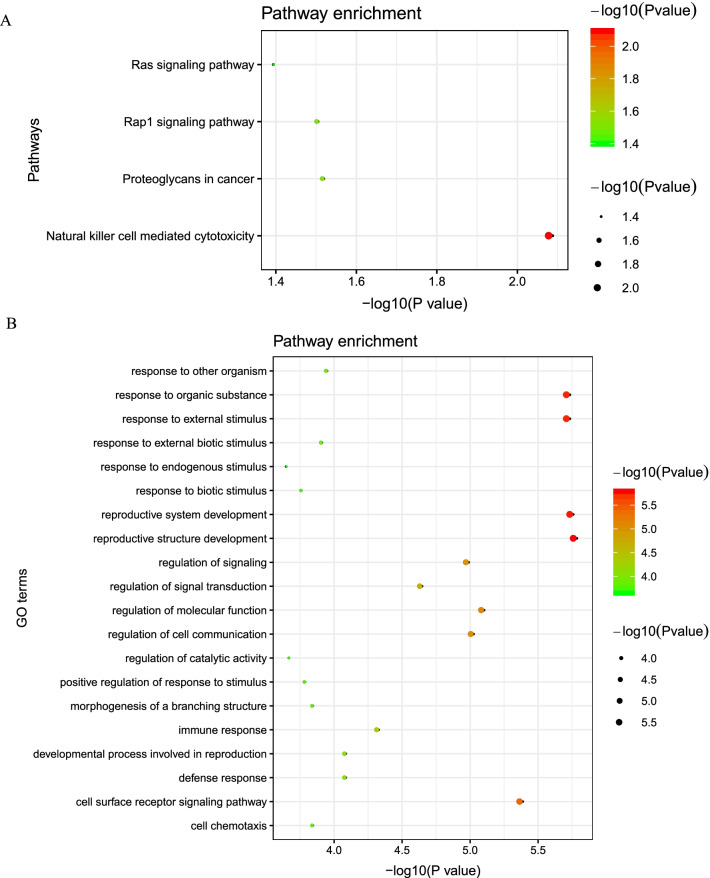
Potential biological functions of the proteins affected by the BMI-associated RNAm-SNPs. **A** KEGG pathway enrichment of the proteins affected by the BMI-associated RNAm-SNPs; **B** The top 20 significant biological process GO terms for the proteins affected by the BMI-associated RNAm-SNPs

We found 54 pQTL signals (*P* < 5.0 × 10^–5^) for 19 RNAm-SNPs that were significantly associated with BMI (*P* < 5.0 × 10^–8^) (Additional file 1: Table S5). Among these signals, 49 were identified in SNPs associated with m^6^A modification. The SNP with the strongest pQTL signal was rs3172494 (m^6^A-associated SNP), which was associated with circulating TXNDC12 levels (*P* = 1.95 × 10^–72^). rs3172494 and rs853678 each were associated with circulating levels of 9 proteins. A total of 50 proteins were associated with BMI-associated RNAm-SNPs. These proteins were enriched in specific KEGG pathways, such as natural killer cell-mediated cytotoxicity (*P* = 8.1 × 10^–3^), proteoglycans in cancer (*P* = 3.0 × 10^–2^), the Rap1 signaling pathway (*P* = 3.1 × 10^–2^) and the Ras signaling pathway (*P* = 4.0 × 10^–2^) (Fig. 3A). These proteins were also enriched in GO terms of biological processes (Fig. 3B).

### Proteins causally associated with BMI

The pQTL analysis showed that RNAm-SNPs were associated with circulating protein levels. To support the functional role of RNAm-SNPs in BMI, we still need to demonstrate that the circulating proteins affected by the RNAm-SNPs were associated with BMI. We chose proteins for MR analysis from two aspects based on the findings of the pQTL analysis. First, 13 proteins (ADH1B, CTSB, DOCK9, MICB, PDE4D, PRDM1, RACGAP1, SCG3, STOM, TDGF1, TMPRSS11D, TNS2 and TXNDC12) that showed strong signals (*P* < 5.0 × 10^–8^) in the pQTL analysis were chosen. Second, five proteins (CCL25, GRIA4, HBEGF, NPPA and RETN) were also considered because they have been reported to be associated with obesity. Therefore, we tested whether these 18 proteins were genetically associated with BMI using four MR methods. Associations with *P* < 6.94 × 10^−4^ were considered significant in this analysis. We found that the associations between circulating levels of seven proteins (ADH1B, DOCK9, MICB, PRDM1, STOM, TMPRSS11D and TXNDC12) and BMI were significant in weighted median, IVW, MR-Egger or MR-PRESSO analyses (Table 3). Based on the results of the MR-Egger and MR-PRESSO analyses, the associations between circulating levels of ADH1B, TMPRSS11D and TXNDC12 and BMI were likely due to pleiotropic effects. Therefore, the MR analyses provided evidence for the causal associations between circulating levels of DOCK9, MICB, PRDM1 and STOM and BMI, and the strongest evidence was found for STOM.

**Table 3:** Association between circulating protein levels and BMI

Proteins	Estimate	Standard Error	*P* values				
			Weighted median	IVW	MR-Egger	Intercept	MR-PRESSO
ADH1B	0.0269	0.0068	8.18E−05	8.00E−02	6.22E−04	9.63E−03	5.06E−01
DOCK9	0.0478	0.0087	4.50E−08	5.44E−03	1.76E−01	5.10E−01	4.98E−02
MICB	-0.0161	0.0039	3.15E−05	4.38E−05	1.18E−02	8.43E−01	1.28E−02
PRDM1	0.0182	0.0034	1.15E−07	4.30E−02	8.09E−02	3.17E−01	3.32E−02
STOM	0.0307	0.0046	3.10E−11	3.87E−06	1.45E−06	5.00E−02	9.89E−03
TMPRSS11D	0.0280	0.0044	2.64E−10	3.27E−02	6.11E−06	2.30E−03	2.48E−02
TXNDC12	−0.0115	0.0029	9.26E−05	3.59E−02	3.57E−01	4.98E−01	8.07E−02

The effect estimation was derived from the weighted median analysis

## Discussion

In this study, 78 RNAm-SNPs associated with BMI were identified by integrating BMI GWAS data with information from the RMVar database and QTL studies. The findings indicated that RNA modification may play a role in obesity. The identified RNAm-SNPs were related to the RNA modifications of m^6^A, m^1^A, m^7^G and A-to-I. These SNPs showed *cis*-acting eQTL effects in relevant tissues, and some of them were found to be associated with proteins that were enriched in specific pathways. Moreover, we demonstrated that the affected gene expression and protein levels were associated with BMI in MR analyses. Therefore, by applying this study strategy, we clarified how RNAm-SNPs affected BMI, i.e., the RNAm-SNPs affect RNA modification, which controls gene expression, and the altered RNA expression or protein levels result in abnormal BMI.

Although hundreds of BMI-related genomic loci have been identified by GWASs, many of the SNPs inside the loci may not be directly causal variants affecting BMI. The genetic associations require more interpretation [35]. Previous studies have applied exome sequencing technologies to detect potential functional variations that can alter amino acid sequences[36]. Functional genetic variants that influence RNA–protein interactions[37] or change the splicing sites of exonic splicing enhancers and silencers [38] through RNA editing [39] have also been identified. Studies have shown that epigenetic factors, such as RNA methylation, may affect the function of RNA and its expression level[16]. Genetic variants affecting RNA modification were potentially functional variants for BMI. Therefore, a combination of information from GWASs and RNA modification database could help determine the causal relationship between gene variants and phenotypes. Our study identified many RNAm-SNPs that were significantly associated with BMI, and some of them were in well-known obesity susceptibility genes. Further eQTL analysis and SMR analysis confirmed that some RNAm-SNPs affect gene expression. For example, BMI-associated SNPs rs6713978 and rs13410999 in *ADCY3* were associated with gene expression of *ADCY3*, and expression levels of *ADCY3* were associated with BMI. *ADCY3* (adenylate cyclase 3) is a protein-coding gene that encodes adenylate cyclase, which is widely distributed in human tissues, especially adipose tissue[40]. It catalyzes the synthesis of ATP into cyclic AMP (cAMP)[41]. The ADCY3-cAMP signaling pathway is known to play a critical role in the regulation of adipogenesis [42]. According to the results of eQTL and SMR analysis, rs6713978 and rs13410999 were associated with the gene expression of *ADCY3* in adipose tissues and blood cells. These two RNAm-SNPs overlap with enhancers in several major tissue types and can alter regulatory motifs (YY1, AP, GR, HDAC2 and Pax). In addition, they were functional loss variants for m^6^A methylation and might affect downstream signaling pathways associated with BMI by influencing the expression levels of *ADCY3*. Therefore, the findings of this study showed that RNAm-SNPs in GWAS-identified BMI loci may be functional variants and that RNAm-SNPs may affect BMI by altering gene expression levels.

In addition, pQTL analysis also found that these RNAm-SNPs affected circulating levels of proteins that were related to obesity. Take MAPK3 and SCG3 as examples. The pQTL analysis showed that rs12716973 and rs12102203 were associated with the protein levels of MAPK3 and SCG3, respectively. The rs12716973 SNP is located in a promoter (chr16:29,936,397–29,939,208) and can alter regulatory motifs and protein binding. SNP rs12102203 is a missense SNP and is located in the binding site of transcription factor CTCF (chr15:51,791,545–51,791,985) and can alter regulatory motifs. MAPK3 (mitogen-activated protein kinase 3), or ERK1, is a very important signaling molecule[43]. Studies have shown that MAPK3 plays a critical role in adipocyte differentiation and obesity and can regulate the formation of fat[44]. Functional enrichment analysis showed that MAPK3 was involved in natural killer cell-mediated cytotoxicity[45–47], proteoglycans in cancer, the Rap1 signaling pathway[48] and the Ras signaling pathway[49, 50] and 124 GO biological process terms, which showed the important role of MAPK3 in obesity[44–50]. SCG3 (secretogranin III) is involved in the regulation of BMI by influencing the secretion of neuropeptides associated with food intake[51]. In addition, the proteins CCL25, GRIA4, HBEGF, NPPA and RETN, which are affected by RNAm-SNPs, have been reported to be related to obesity. More importantly, circulating levels of DOCK9, MICB, PRDM1 and STOM were causally associated with BMI in MR analysis. The relationships between these identified proteins and obesity have not been studied. The MR analysis identified risk factors for obesity, and the results suggested that genes involved in natural killer cell-mediated cytotoxicity, proteoglycans in cancer, the Rap1 signaling pathway and the Ras signaling pathway play functional roles in obesity. In summary, the results indicated that these RNAm-SNPs may be involved in the pathogenesis of obesity by changing the protein levels.

The present study has some potential limitations. First, most of the identified RNAm-SNPs were related to m^6^A methylation. Information for other types of RNA modification is lacking, so very few associations between other types of RNA modification and BMI have been identified. Second, we did not test whether the identified RNAm-SNPs functionally affected the RNA modifications experimentally. RNA modification QTL studies are scarce at present. We looked for a m^6^A QTL for BMI-associated RNAm-SNPs in the literature and found that only one m^6^A-SNP, the missense SNP rs4858871 in *MAP4*, was a m^6^A QTL. This SNP was associated with the methylation level of the m^6^A peak chr3_47916105_47916283 in muscle and heart tissues[19]. Third, the relationships between protein molecules and BMI have not been verified experimentally. However, the identification of related proteins was performed to find evidence to support the functional relevance of the identified RNAm-SNPs in obesity. This purpose was achieved by applying MR analysis to establish the potential causal associations between proteins and BMI.

## Conclusions

In summary, associations between RNAm-SNPs and BMI were identified by mining GWAS datasets in this study. Our study suggested that RNAm-SNPs may affect BMI by altering gene expression. Gene expression levels (e.g., *ADCY3*) and circulating protein levels (e.g., DOCK9, MICB, PRDM1 and STOM) affected by the RNAm-SNPs were associated with BMI. Therefore, RNA modification of these genes may be an important regulatory mechanism of BMI. This is the first attempt to clarify the relationship between RNAm-SNPs, gene expression and BMI.

## Supplementary Information

**Additional file 1. Table S1.** RNAm_SNPs identified for BMI. **Table S2.** Replications of associations between RNAm_SNPs and BMI in East Asian population. **Table S3.** Associations between RNAm-SNPs and gene expression levels in adipose tissues. **Table S4.** Associations between gene expressions in different tissues and BMI. **Table S5** Associations between BMI-associated RNAm-SNPs and plasma protein levels.

## Data Availability

The dataset(s) supporting the conclusions of this article is(are) included within the article (and its additional file(s)).
